# Genetic Polymorphisms of TYMS, MTHFR, ATIC, MTR, and MTRR Are Related to the Outcome of Methotrexate Therapy for Rheumatoid Arthritis in a Chinese Population

**DOI:** 10.3389/fphar.2018.01390

**Published:** 2018-11-28

**Authors:** Shuang Lv, HuiZhen Fan, Jiang Li, Hui Yang, Jing Huang, XiaoMing Shu, Lu Zhang, Yuan Xu, Xiaoya Li, Jieyu Zuo, Cheng Xiao

**Affiliations:** ^1^Institute of Clinical Medicine, China-Japan Friendship Hospital, Beijing, China; ^2^Beijing University of Chinese Medicine, Beijing, China; ^3^Department of Gastroenterology, People’s Hospital of Yichun, Yichun, China; ^4^Department of Laboratory Medicine, China-Japan Friendship Hospital, Beijing, China; ^5^Department of Rheumatology, China-Japan Friendship Hospital, Beijing, China; ^6^Department of TCM Rheumatology, China-Japan Friendship Hospital, Beijing, China; ^7^Chinese Academy of Medical Sciences and Peking Union Medical College, Beijing, China; ^8^Faculty of Pharmacy and Pharmaceutical Sciences, University of Alberta, Edmonton, AB, Canada

**Keywords:** methotrexate, rheumatoid arthritis, single nucleotide polymorphisms, TYMS, MTHFR, ATIC, MTR, MTRR

## Abstract

**Objective:** Analysis of the relationship between single nucleotide polymorphisms (SNPs) and outcomes of methotrexate (MTX) therapy for rheumatoid arthritis (RA) in China.

**Materials and Methods:** TYMS 28 bp VNTR (rs34743033), MTHFR [677C>T (rs1801133) and 1298A>C (rs1801131)], ATIC 347C>G (rs2372536), MTR A2756G (rs1805087), and MTRR 66A>G (rs1801394) enzyme proteins may be related to the outcomes of MTX therapy, according to our previous meta-analysis. A total of 162 patients with RA were included in our study. SNPs were evaluated using polymerase chain reaction (PCR). Disease Activity Score 28 (DAS28) was used to evaluate the clinical response, and adverse drug reactions (ADRs) were collected after physical examinations of the patients.

**Results:** The MTHFR 677C>T gene showed a relationship with the ADRs of MTX in the Recessive model [TT vs. (CC+CT)] (*p* = 0.04, OR = 2.20, 95% CI: 1.01, 4.77). In the Codominant model [CT vs. (CC+TT)], the MTHFR 677C>T gene also showed a trend of association with ADRs (*p* = 0.08, OR = 0.52, 95% CI: 0.25, 1.08). No significant difference was found between TYMS, MTHFR, ATIC, MTR, and MTRR gene polymorphisms and the RA response or ADRs related to MTX in our study.

**Conclusion:** Our results showed that the MTHFR [677C>T (rs1801133)] TT genotype is associated with ADRs to MTX in Chinese RA patients. Other SNPs, including TYMS 28bp VNTR (rs34743033), MTHFR [677C>T (rs1801133) and 1298A>C (rs1801131)], ATIC 347C>G (rs2372536), MTR A2756G (rs1805087), and MTRR 66A>G (rs1801394) gene polymorphisms, were not associated with MTX treatment outcomes. Further studies are required to validate these findings.

## Introduction

Rheumatoid arthritis (RA) is characterized by synovial inflammation, cartilage damage and bone erosion (Walter et al., 2016; Fessler et al., 2017). The pathogenesis of RA, which is a systemic inflammatory disease, remains unclear. Additionally, curative therapy for RA has not been established. Currently, disease-modifying anti-rheumatic drugs (DMARDs) are usually used to delay symptom progression and achieve pain relief (Kojima et al., 2016). One DMARD, methotrexate (MTX), is widely used worldwide to treat RA because it is inexpensive, effective and safe (Swierkot et al., 2015); however, 30–40% of patients on MTX therapy fail to attain remission (Ranganathan and McLeod, 2006), and 30–40% of patients have problems with safety and tolerability (Wevers-de Boer et al., 2012). Six single nucleotide polymorphisms (SNPs) that encode proteins involved in MTX metabolism are examined in our meta-analysis (Qiu et al., 2017a,b).

Methotrexate has 1 glutamate moiety, so it is also referred to as MTX polyglutamate (MTXPG1). MTX polyglutamates (MTXPGs) can persist in the long term inside the target cell, and folylpolyglutamate synthase (FPGS) converts MTX into MTXPGs in the cells. After gamma-glutamyl hydrolase (GGH) converts MTXPGs to MTX by removing the glutamates, the drug is removed from the cells (Yamamoto et al., 2016).

Methotrexate polyglutamates inhibit thymidylate synthase (TYMS), which is a key protein involved in the *de novo* pyrimidine synthesis pathway. Methylenetetrahydrofolate reductase (MTHFR) is also a key enzyme in the *de novo* synthesis pathway (Song et al., 2014). TYMS competes with MTHFR for one of its substrates: 5,10-MTHF (Chaabane et al., 2018). MTXPGs can inhibit aminoimidazole carboxamide ribonucleotide transformylase (ATIC), causing the intracellular accumulation of aminoimidazole carboxamide ribonucleotide, which in turn inhibits adenosine-metabolizing enzymes. MTXPGs also directly inhibit methionine synthase (MTR) and methionine synthase reductase (MTRR) (Kato et al., 2012).

The aims of our study were to determine the correlation of the TYMS 28bp VNTR (rs34743033), MTHFR [677C>T (rs1801133) and 1298A>C (rs1801131)], ATIC 347C>G (rs2372536), MTR A2756G (rs1805087) and MTRR 66A>G (rs1801394) polymorphisms with the clinical response and adverse drug reactions (ADRs) to MTX treatment in Chinese RA patients.

## Materials and Methods

### Materials

This was a retrospective study conducted between April 2016 and April 2018 at China-Japan Friendship Hospital and People’s Hospital of Yichun of a cohort of consecutive Chinese Han patients with RA treated with MTX. A total of 162 patients from 23 provinces were included in our study. All the patients who were selected had to meet the 2010 revised classification criteria of the American College of Rheumatology and European League against Rheumatism (Aletaha et al., 2010). Each patient had been treated with MTX (mean dose = 8.92 ± 2.26 mg/week) for at least 3 months. DAS28 was used to evaluate the MTX treatment response (Versteeg et al., 2018). The DAS28 of RA patients in our study was 5.17 ± 1.5. And the mean duration of RA patients was 4 years. Patients were excluded if they had recently been pregnant or desired to become pregnant. All of this study’s protocols involving human subjects were approved by the ethics committee of People’s Hospital of Yichun (ethics ID: 2014-01). Written informed consent was obtained from each participant.

We also recorded analytical data, such as body mass index (BMI), erythrocyte sedimentation rate (ESR), C-reaction protein (CRP), rheumatoid factor (RF), cyclic citrullinated peptide (CCP), antinuclear antibody (ANA), and anti-keratin antibody (AKA) levels.

### Methods

#### DNA Extraction

For the genetic analysis, 2 ml of peripheral blood was obtained from each patient using the standard venipuncture technique. Each sample was centrifuged, and the white blood cells were separated by pipette. The samples were stored at -80°C. DNA was extracted using the QIA amp DNA Blood Mini Kit according to the QIAGEN manufacturer’s instructions (Hilden, Florida, Germany). Quality control procedures were performed using a ND-1000 Spectrophotometer by NanoDrop Technologies (Wilmington, DE, United States) to ensure the sample and polymorphism genotyping success rates before analysis by requiring that the 260/280 was between 1.8 and 1.9 (Owen et al., 2013). We determined the precise length of the genomic DNA by gel electrophoresis using 1% agarose gel. All the purified samples were stored at -80°C until the next analyses.

#### Allele Genotyping

The gene polymorphisms were produced by polymerase chain reaction (PCR) and confirmed through sequencing. The forward and reverse primers were designed by Primer 5 and are described in Table 1. Six SNPs were examined in this study. All polymorphisms were genotyped using sequencing. PCR reactions were performed using 3 μl of 10x PCR reaction buffer (Osaka, Kansai, Japan), 5 μl of 100 mmol/L dNTP (Osaka, Kansai, Japan), 3 μl of each primer at a concentration of 10 μM, 14 μl of Rnase- and DNase-free water (Osaka, Kansai, Japan), and 2 μl of sample DNA at a total volume of 30 μl per single-well reaction. Assay conditions were 3 min at 94°C, 30 s at 58°C, 30 s at 94°C and 40 cycles of 72°C for 1 min. Then it was kept at 72°C for 10 min. Chromas software version 2.23 (Brisbane, QLD, Australia) was used as an absolute quantification assay to analyze the SNP assay. Each of the MTHFR677C>T (rs1801133) genes and TYMS 28bp VNTR (rs34743033) had another genetic polymorphisms, and we abandoned these data when analyzing the relationship between the gene polymorphisms and MTX treatment outcomes.

**Table 1:** Gene primers.

Gene	Forward primer	Reverse primer
TYMS	GCTCCGTTCTGTGCCACACC	GGCGTCACCTCTCAGGCTGT
MTHFR677	AGGTCTCCCAACTTACCCTTCT	GCTTGTGGTTGACCTGGGAGGA
MTHFR1298	CAGTCATGAGCCCAGCCACTCACT	ACTCAGCGAACTCAGCACTCCACC
ATIC	ATGTCAGGCTCACAGTTTATAT	CAAAACACAATCCAGAAGTAGC
MTR	TGTTATCAGCATTGACCATTAC	TGAAGACCTCTGATTTGAACTA
MTRR	GCTTGTCTACAGGGTTGCACTTAG	TACAGTGAAGATCTGCAGAA

### Statistical Analysis

Ethnic and gender differences in the distribution of allelic frequencies among genotypes were tested using the Hardy–Weinberg equilibrium. A *p*-value of <0.05 was considered to represent a significant difference in all statistical analyses. All statistical data are described as numbers and frequencies. Depending on data distribution, Student’s *t*-test or Mann–Whitney’s *U*-test was used to analyze continuous variables. Means and standard deviations (±SD) or interquartile ranges (IQRs) were used to describe the sample. Allele and genotype risk was assessed using odds ratios (ORs) and 95% confidence interval values (CIs) based on the chi-square test. When the expected count was less than 5, we used the Fisher chi-square test instead of the chi-square test. Statistical analysis was performed with Statistical Package for Social Sciences (SPSS) Version 20 (Armonk, NY, United States).

The gene polymorphisms were analyzed using five models; for example, MTRR 66A>G (rs1801394) was analyzed with the Pre-allele model (A vs. G), the Dominant model [AA vs. (AG+GG)], the Recessive model [GG vs. (AG+AA)], the Codominant model [AG vs. (AA+GG)] and the Homozygotic model (AA vs. GG).

## Results

### Description of the Population

A total of 162 RA patients were observed in this study, and the characteristics of the RA group are presented in Table 2. All the patients were treated with MTX for at least 3 months. Our results showed no differences in terms of age, sex, DAS28 results, MTX dose, treatment duration, ESR, CPR, RF positive status, CCP positive status, AKA positive status, ANA positive status, individual variables of DAS28 and smoking and drinking habits between the responder and non-responder groups.

**Table 2:** Characteristics of the study population.

Characteristics	RA patients (*n* = 162)	Responders (*n* = 99)	Non-responders (*n* = 63)	*P*-value
Age (years)	52.99 ± 13.81	52.83 ± 13.94	53.25 ± 13.71	0.849
Gender (female/male)	136/26	79/20	57/6	0.288
Duration (years)	4 (2.10)	4 (2.10)	5 (2.10)	0.345
Dose of MTX (mg/week)	10 (7.5.10)	10 (7.5.10)	8.75 (7.5.10)	0.396
CRP (mg/dl)	1.49 (0.64.3.99)	1.42 (0.59.3.95)	1.54 (0.64, 4.30)	0.626
ESR (mm/h)	30 (16.75.59.50)	25 (16.00.50.00)	33 (20.00, 69.00)	0.056
RF positive	95.95% (142/148)	94.38% (84/89)	98.31% (58/59)	0.403
CCP positive	82.84% (111/134)	85.54% (71/83)	78.43% (40/51)	0.289
AKA positive	54.87% (62/113)	51.47% (35/68)	69% (27/45)	0.372
ANA positive	58.87% (73/124)	55.26% (42/76)	64.68% (31/48)	0.304
DAS28-ESR	5.17 ± 1.50	5.18 ± 1.50	5.17 ± 1.57	0.973
BMI KG/M2	23.09 (20.70.25.53)	23.56 (21.30.25.88)	22.31 (19.92,24.93)	0.063
Smoking Drinking	9.88% (16/162)	12.12% (12/99)	6.35% (4/63)	0.230
Drinking	7.41% (12/162)	6.06% (6/99)	9.52% (6/63)	0.189

*n, number of patients; response > 0.6 change in DAS28 before and after MTX treatment; response ≤ 0.6 change in DAS28. Values are presented as the mean ± SD or median (interquartile range) or percent.*

### Frequencies and Polymorphisms of TYMS, MTHFR, ATIC, MTR, and MTRR

Among the 162 RA patients, 99 RA patients responded to MTX therapy, and 63 RA patients did not. During MTX therapy, 39 RA patients among 162 RA patients developed ADRs, as illustrated in Table 3. Gastrointestinal reactions (abdominal and stomach pain, diarrhea, nausea and indigestion or anorexia) were the most frequent events (29/162 patients, 17.90%); hepatic reactions were less frequent (12/162 patients, 7.41%). Interstitial lung disease, pain throughout the body, erythema of the extremities, hair loss and vertigo reactions were the least frequent ADRs, 0.61% (1/162 patients). Adverse drug events were observed after 3 months or 6 months.

**Table 3:** Adverse drug reactions (ADRs) observed in 162 RA patients.

	ADRs	Frequency, *n* (%)
None	39	24.07%
General	Tiredness	6 (3.70%)
	Pain	1 (0.61%)
	Vertigo	1 (0.61%)
	Nausea	11 (6.79%)
	Stomach pain	8 (4.94%)
	Abdominal pain	6 (3.70%)
	Indigestion or anorexia	2 (1.23%)
	Diarrhea	2 (1.23%)
	Hair loss	1 (0.61%)
	Erythema of the extremities	1 (0.61%)
	Liver disease	12 (7.41%)
	Interstitial lung disease	1 (0.61%)

Table 4 presents a comparison of the distribution of MTX-related enzyme gene polymorphisms according to the Hardy–Weinberg equilibrium. All *p*-values for the chi-square test for agreement with the Hardy–Weinberg equilibrium had no statistical significance (*P* < 0.05). Table 5 presents the TYMS, MTHFR, ATIC, MTR, and MTRR gene polymorphism distributions of responders and non-responders. Our results showed no significant difference in the frequency distribution of genotypes, alleles, and haplotypes of TYMS 28bp VNTR (rs34743033), MTHFR [677C>T (rs1801133) and 1298A>C (rs1801131)], ATIC 347C>G (rs2372536), MTR A2756G (rs1805087) and MTRR 66A>G (rs1801394) in one case using all five statistical models (the Pre-allele model, Dominant model, Recessive model, Codominant model, and Homozygotic model).

**Table 4:** Comparison of the distribution of MTX-related enzyme gene polymorphisms.

Ethnic/ population group	*n*	Genotype frequency (*n*)			HWE	Allele frequency		*P*-value
TYMS	161	3R3R	3R2R	2R2R	1. 17	3R	2R	0.28
		63.35%	34.16%	2.48%		80.43%	19.57%	
		(102)	(55)	(4)		(259)	(63)	
MTHFR 677	161	CC	CT	TT	2.53	C	T	0.11
		18.01%	55.90%	26.09%		45.96%	54.04%	
		(29)	(90)	(42)		(148)	(174)	
MTHFR 1298	162	AA	AC	CC	6.77	A	C	0.99
		70.99%	26.54%	2.47%		84.26%	15.74%	
		(115)	(43)	(4)		(273)	(51)	
ATIC 347	162	CC	CG	GG	0.01	C	G	0.92
		59.88%	35.19%	4.94%		77.47%	22.53%	
		(97)	(57)	(8)		(251)	(73)	
MTR 2756	162	AA	AG	GG	0.01	A	G	0.93
		78.40%	32.77%	1.23%		88.58%	11.42%	
		(127)	(33)	(2)		(287)	(37)	
MTRR 66	162	AA	AG	GG	1.02	A	G	0.31
		61.11%	35.80%	3.09%		79.01%	20.99%	
		(99)	(58)	(5)		(256)	(68)	

*HWE: P-value for chi-square test for agreement with the Hardy–Weinberg equilibrium.*

**Table 5:** Genotype and allele frequencies in responders and non-responders.

Genotypes or alleles	Response	Non-response	*P*-value^a^	Compared	*P*-value^b^	OR (95% CI)
	(*n* = )	(*n* = )		genotypes or alleles		
TYMSVNTR						
rs34743033 genotype						
3R3R	58	44		3R3R vs. 3R3R+3R2R	0.17	0.63 (0.32, 1.23)
3R2R	37	18		2R2R vs. 3R2R+3R3R	1	1.96 (0.20, 19.25)
2R2R	3	1	0.46	3R2R vs. 2R2R+3R3R	0.23	1.52 (0.77, 3.00)
Allele						
3R	153	106		3R3R vs. 2R2R	0.64	0.44 (0.04, 4.37)
2R	43	20	0.18			
MTHFR 677C>T						
rs1801133 genotype						
CC	19	10		CC vs. CT+TT	0.57	1.28 (0.55, 2.96)
CT	51	39		TT vs. CC+CT	0.37	1.40 (0.67, 2.93)
TT	28	14	0.46	CT vs. CC+TT	0.22	0.67 (0.35, 1.27)
Allele						
C	89	59		CC vs. TT	0.30	0.57 (0.20,1.65)
T	107	67	0.80			
MTHFR 1298A>C						
rs1801131 genotype						
AA	68	47		AA vs. AC+AA	0.42	0.75 (0.37, 1.52)
AC	29	14		CC vs. AC+CC	0.64	0.63 (0.09, 4.58)
CC	2	2	0.55	AC vs. AA+CC	0.32	1.45 (0.70, 3.02)
Allele						
A	165	108		AA vs. CC	1	1.45 (0.20, 10.64)
C	33	18	0.57			
ATIC 347C>G						
rs2372536 genotype						
CC	61	36		CC vs. CG+GG	0.57	1.20 (0.63, 2.29)
CG	34	23		GG vs. CG+CC	0.71	0.62 (0.15, 2.58)
GG	4	4	0.71	CG vs. CC+GG	0.78	0.91 (0.47, 1.76)
Allele						
C	156	95		CC vs. GG	0.48	1.69 (0.40, 7.19)
G	42	31	0.48			
MTR 2756A>G						
rs1805087 genotype						
AA	76	51		AA vs. AG+GG	0.53	0.78 (0.36, 1.70)
AG	21	12		GG vs. AG+AA	0.52	1.65 (1.46, 1.87)
GG	2	0	0.67	AG vs. AA+GG	0.74	1.14 (0.52, 2.53)
Allele						
A	173	114		AA vs. GG	0.52	0.59 (0.52, 0.69)
G	25	12	0.39			
MTRR 66A>G						
rs1801394 genotype						
AA	61	38		AA vs. AG+GG	0.87	1.06 (0.55, 2.02)
AG	36	22		GG vs. AG+AA	0.38	0.41 (0.07, 2.54)
GG	2	3	0.62	AG vs. AA+GG	0.85	1.06 (0.55, 2.06)
Allele						
A	158	98		AA vs. GG	0.38	2.41 (0.39, 15.08)
G	40	28	0.66			

*^a^ P-value for chi-square test or Fisher chi-square test for responders and non-responders in different gene and Pre-allele models; ^b^ P-value for chi-square test for responders and non-responders in the Dominant model, Recessive model, Codominant model, and Homozygotic model. When the expected count was less than 5, we used the Fisher chi-square test instead of the chi-square test.*

Table 6 presents the frequency distribution of TYMS 28bp VNTR (rs34743033), MTHFR [677C>T (rs1801133) and 1298A>C (rs1801131)], ATIC 347C>G (rs2372536), MTR A2756G (rs1805087) and MTRR 66A>G (rs1801394) polymorphisms in patients with and without ADRs. Only one SNP, MTHFR 677C>T (rs1801133), among the five genes was associated with an increased risk of MTX ADRs in our study. In the Recessive model [TT vs. (CC+CT)], the *p*-value was 0.04 (OR = 2.11, 95% CI: 1.01, 4.77). In the Codominant model [CT vs. (CC+TT)], the *p*-value was 0.08 (OR = 0.52, 95% CI: 0.25, 1.08), which showed a trend of association. The incidence of adverse effects in another Chinese study (Xiao et al., 2010) was 49.5% (47/95), but in our study, it was 24.07% (39/162). However, the same result was found. In the other study, the *p*-value of ADRs and MTHFR 677C>T (rs1801133) was 0.01, and the T allele was 0.009. Although the *p*-values in our study were 0.11 and 0.21, the TT genotype was 0.04. Both studies showed that MTHFR 677C>T (rs1801133) was associated with adverse effects (*p* < 0.05), and MTHFR 1298A>C (rs1801131) was not (*p* > 0.05). It is possible that the MTHFR 677C>T (rs1801133) but not the MTHFR 1298A>C (rs1801131) gene was associated with adverse effects in the Chinese population with RA.

**Table 6:** Genotype and allele frequencies in ADR and non-ADR groups.

Genotypes or alleles	ADRs	Non-ADRs	*P*-value^a^	Compared	*P*-value^b^	OR (95% CI)
	(*n* = )	(*n* = )		genotypes or alleles		
TYMS VNTR						
rs34743033 genotype						
3R3R	25	77		3R3R vs. 3R3R+3R2R	0.91	1.04 (0.49, 2.21)
3R2R	13	42		2R2R vs. 3R2R+3R3R	1	1.04 (0.11, 10.33)
2R2R	1	3	1	3R2R vs. 2R2R+3R3R	1	0.95 (0.44, 2.04)
Allele						
3R	63	196		3R3R vs. 2R2R	1	0.97 (0.10, 9.79)
2R	15	48	0.93			
MTHFR677C>T						
rs1801133 genotype						
CC	7	22		CC vs. CT+TT	0.58	1.38 (0.53, 3.63)
CT	17	73		TT vs. CC+CT	0.04	2.20 (1.01, 4.77)
TT	15	27	0.11	CT vs. CC+TT	0.08	0.52 (0.25, 1.08)
Allele						
C	31	117		CC vs. TT	0.30	0.57 (0.20, 1.65)
T	47	127	0.21			
MTHFR1298A>C						
rs1801131 genotype						
AA	28	87		AA vs. AC+AA	0.9	1.05 (0.48, 2.34)
AC	11	32		CC vs. AC+CC	0.57	1.33 (1.21, 1.45)
CC	0	4	0.75	AC vs. AA+CC	0.79	1.12 (0.50, 2.50)
Allele						
A	67	206		AA vs. CC	0.57	0.76 (0.68, 0.84)
C	11	40	0.65			
ATIC347C>G						
rs2372536 genotype						
CC	26	71		CC vs. CG+GG	0.32	1.47 (0.69, 3.12)
CG	12	45		GG vs. CG+CC	0.68	0.44 (0.05, 3.66)
GG	1	7	0.63	CG vs. CC+GG	0.51	0.77 (0.36, 1.67)
Allele						
C	64	187		CC vs. GG	0.68	2.56 (0.30, 21.85)
G	14	59	0.48			
MTR2756A>G						
rs1805087 genotype						
AA	32	95		AA vs. AG+GG	0.52	1.35 (0.54, 3.38)
AG	7	26		GG vs. AG+AA	1	1.32 (1.21, 1.44)
GG	0	2	0.67	AG vs. AA+GG	0.67	0.82 (0.32, 2.06)
Allele						
A	71	216		AA vs. GG	1	0.75 (0.68, 0.83)
G	7	30	0.44			
MTRR 66A>G						
rs1801394 genotype						
AA	22	77		AA vs. AG+GG	0.59	0.77 (0.37, 1.61)
AG	17	41		GG vs. AG+AA	0.34	1.33 (1.22, 1.46)
GG	0	5	0.31	AG vs. AA+GG	0.25	1.55 (0.74, 3.23)
Allele						
A	61	195		AA vs. GG	0.58	-
G	17	51	0.84			

*^a^ P-value for the chi-square test or Fisher chi-square test for the ADRs and non-ADR groups in the different gene and Pre-allele models; ^b^ P-value for chi-square test for the ADR and non-ADR groups in the Dominant model, Recessive model, Codominant model, and Homozygotic model. When the expected count was less than 5, we used the Fisher chi-square test instead of the chi-square test.*

## Discussion

### Analysis of TYMS, MTHFR, ATIC, MTR, and MTRR Polymorphisms Related to the Clinical Efficacy of MTX

Thymidylate synthase is located on chromosome 18q11.32 and is essential for the simultaneous conversion of deoxyuridine monophosphate (dUMP) and 5,10 methylenetetrahydrofolate (5,10-MTHF) to thymidylate monophosphate (dTMP) and dihydrofolate (DHF) (Lima et al., 2014b). Then, dTMP is phosphorylated to deoxythymidine triphosphate (dTTP), which is used for deoxyribonucleic acid (DNA) synthesis and repair (Lima et al., 2013). 5,10-methyl-THF is required for purine and pyrimidine synthesis. The promoter-enhancer region of the TYMS gene contains a double (2R) or a triple (3R) 28-base-pair (28-bp) tandem repeat polymorphism (Corre and Galibert, 2005). The enzymatic activity of TYMS increases with an increasing number of repeated 28-bp sequences because a putative Enhancer box (E-box) sequence is exhibited on the first 28-bp repeat of the 2R allele and on the first two repeats of the 3R allele (Lincz et al., 2007). In the current study, TYMS 2R/3R and 6-bp I/D polymorphisms may not be associated with non-responsiveness to or toxicity of MTX therapy in RA patients (Bae and Lee, 2018). However, Seabra Lima’s study (Lima et al., 2014a) of 233 Caucasian RA patients treated with MTX revealed that the TYMS genotype 3R3R (*p* = 0.005, OR = 2.34) was associated with non-response to MTX. TYMS gene polymorphisms were not related to MTX treatment outcomes in south Indian Tamil patients with RA (Muralidharan et al., 2017), and the same results were observed in our study.

The MTHFR enzyme is responsible for several crucial cellular processes, and its deficiency can have many consequences for folate status, which can influence the clinical response to MTX treatment (Jekic et al., 2013). In the case of MTHFR, a C is transited to a T in exon 4 of the SNP rs1801133, which entails the change of an alanine for a valine in position 222 of the protein; additionally, an A is transited to a C in exon 7 of the SNP rs1801131, which provokes the change of a glutamine for an alanine in position 429 of the protein (Sala-Icardo et al., 2017). Boughrara et al. (2017) showed that MTHFR [677C>T (rs1801133) and 1298A>C (rs1801131)] gene polymorphisms were not associated with the efficacy of MTX treatment in West Algerian RA patients. In south Asia, Indian RA patients with a MTHFR 1298 A allele were related to those with MTHFR 1298 CC (OR: 2.6; 95% CI: 1.1–5.8; *p* = 0.02) (Ghodke-Puranik et al., 2015). One meta-analysis showed that MTHFR 1298 gene polymorphisms were not associated with the outcome of MTX treatment in RA patients (Fan et al., 2017), while another meta-analysis indicated that MTHFR C677T and A1298C polymorphisms could be predictors of the risk of RA. However, data are currently insufficient to definitively confirm or refute the association between MTHFR gene polymorphisms and RA. Xiao et al. (2010) found that the MTHFR 677C>T (rs1801131) gene polymorphisms was associated with the clinical response to MTX, while MTHFR 1298A>C (rs1801133) was not. All 110 RA patients in his study were Chinese Han people from Anhui Province. In our study, neither MTHFR 677C>T (rs1801133) nor 1298A>C (rs1801131) was related to the efficacy MTX treatment for RA. The clinical baseline characteristics in Xiao’s study were similar to those of our study and included age, sex, and dose of MTX. However, in our study, the RA patients were from 23 provinces representing approximately 67.65% of China (23/34). It is possible that the inclusion of RA patients from different provinces affected the outcomes, and the inclusion of a larger samples would permit a more definite conclusion regarding the relationship between the MTHFR 677C>T gene polymorphisms and ADRs to MTX in China.

Additionally, intracellular MTXPGs are known to show greater binding affinity for ATIC compared with MTX (Baggott et al., 1986). The ATIC gene is located at chromosome 2q35, and the most common genetic polymorphism investigated is ATIC 347C>G (rs2372536) on exon 5, which results in threonine to serine substitution at position 116 of the gene (Hinks et al., 2011). AICAR will accumulate inside the cells when ATIC is inhibited, causing the release of adenosine into the extracellular space. Adenosine exerts anti-inflammatory effects by reducing neutrophil adherence and inhibiting the activity of natural killer cells, monocyte macrophages and T lymphocytes (Cronstein, 2005). Studies have reported that the ATIC rs2372536 GG genotype is associated with non-response to MTX in Caucasian patients (OR: 2.40; 95% CI: 1.30, 4.42; *p* = 0.005) (Kurzawski et al., 2016), but not in south Indian populations (OR: 0.98; 95% CI: 0.54–1.77; *p* = 0.95) (Lee and Bae, 2016; Muralidharan et al., 2016). A similar result was obtained in our present study, which showed that ATIC rs2372536 was not related MTX treatment outcomes in RA patients.

The MTR and MTRR genes play pivotal roles in both homocysteine metabolism (Leclerc et al., 1996) and folate metabolism (Muralidharan et al., 2018). The MTR gene uses the methyl group from 5-MTHF to remethylate homocysteine. The products of this reaction are methionine and tetrahydrofolate (THF) (Nikbakht et al., 2012). MTR is located at 1q 43 and is essential for maintaining adequate intracellular methionine, intracellular folate, and normal homocysteine concentrations (Leclerc et al., 1996). A polymorphism in the MTR gene A2756G (rs1805087) may decrease enzyme activity (Jacques et al., 2003). The MTRR gene is located on chromosome 5p15.3 and encodes for the enzyme MTRR, which is involved in the reductive regeneration of cob (I) alamin (Jekic et al., 2013). In the Netherlands, no associations between the MTRR and MTR gene polymorphisms and good clinical responses were found in recent-onset RA patients (Wessels et al., 2006). A study (Gonzalez-Mercado et al., 2017) of 110 Mexican RA patients who were diagnosed at least 1 year previously and had been receiving MTX for at least 3 months showed no association between MTRR gene polymorphism and MTX efficacy. Another study (Lopez-Rodriguez et al., 2018) of European Caucasian RA patients showed that MTRR fulfilled the high association standards. In Japan, Kato et al. (2012) showed that RA patients with the GG genotype at MTR A2756G (rs1805087) had a significantly higher total concentration of MTX-PGs in the red cells. This result may indicate that MTR gene polymorphisms are related to the clinical response to MTX, but there was no relationship between them either in South Indian patients (Muralidharan et al., 2018) or in our sample.

### Analysis of TYMS, MTHFR, ATIC, MTR, and MTRR Polymorphisms Related to Adverse Drug Reactions to MTX

Toxicity: MTX-related adverse reactions were defined as the presentation of ADRs related to MTX at each visit. We classified the type of ADRs into System Organ Class (SOC) disorders in accordance with Common Terminology Criteria for Adverse Events (CTCAE) (Chaabane et al., 2016).

It has been reported that TYMS 28bp VNTR (Baggott et al., 1986), MTHFR 677C>T (Berkun et al., 2004; Caliz et al., 2012; Berkani et al., 2017), MTHFR 1298A>C (Berkun et al., 2004; Davis et al., 2014; Berkani et al., 2017), ATIC 347C>G (Muralidharan et al., 2016; Hakamata et al., 2018), MTR A2756G (Nikbakht et al., 2012) and MTRR 66A>G (Dervieux et al., 2006) may be associated with MTX toxicity. A study of 273 Caucasian patients with RA treated with MTX for at least 6 months showed that there were no associations between TYMS gene polymorphisms and the toxicity of MTX treatment (Swierkot et al., 2015). Another study of 185 Tunisian patients, including 35 men and 136 women with RA, showed that MTHFR A1298C, TYMS and MTR A2756G gene polymorphisms all had no association with adverse reactions to MTX (Yamamoto et al., 2016). In Spain, Salazar et al. (2014) found that two SNPs in the ATIC gene (rs16853826 and rs10197559) were associated with toxicity, but rs2372536 was not. Caliz et al. (2012) found that the MTR A2756G and MTRR A66G gene polymorphisms were not associated with increased MTX toxicity. The relationship between gene polymorphisms and ADRs to MTX were inconsistent among populations from different geographic locations.

Whether the TYMS 28bp VNTR (rs34743033), MTHFR 1298A>C (rs1801131), ATIC 347C>G (rs2372536), MTR A2756G (rs1805087), and MTRR 66A>G (rs1801394) polymorphisms are associated with ADRs to MTX, as we found, needs to be confirmed with larger samples.

## Conclusion

Our results represent the first report regarding the relationship between the genetic polymorphisms of TYMS, MTHFR, ATIC, MTR, and MTRR related to the therapeutic outcome of MTX for RA in the Chinese population. Only the MTHFR 677C>T TT genotype was associated with ADRs to MTX. The strength of our study was that our patients came from all over China. However, the limitation of our study was that not all the functionally relevant genetic variants in TYMS, MTHFR, ATIC, MTR, and MTRR were included in our study, and the patient sample in our study was not large enough. Therefore, further studies with a larger sample are needed to confirm our findings.

## Author Contributions

CX formulated the concept and designed the paper. SL, HY, HF, XS, LZ, JZ, JH, YX, and XL conducted the experiments. CX, HF, and HY supplied critical reagents of QIA amp DNA Blood Mini Kit and designed the primers of gene. JL contributed statistical analysis. CX and SL wrote the manuscript.

## Conflict of Interest Statement

The authors declare that the research was conducted in the absence of any commercial or financial relationships that could be construed as a potential conflict of interest.
